# Effectiveness of Echinochrome on HFD-Induced Hyperlipidemia in Rats

**DOI:** 10.1007/s13659-019-00221-4

**Published:** 2019-10-18

**Authors:** Sohair R. Fahmy, Nashwah Ismail Zaki, Shaimaa Zakaria Eid, Ayman Saber Mohamed, Sarah S. Hassanein

**Affiliations:** 1 Zoology Department, Faculty of Science, Cairo University, Giza, 12613 Egypt; 2 Physiology Department, National Organization for Drug Control and Research, Cairo, Egypt

**Keywords:** High-fat diet, Atorvastatin, Echinochrome, Obesity, Oxidative stress

## Abstract

Obesity has been identified with an expanded danger of a progression of illnesses that include different organ-frameworks of the body. In the present examination, we evaluated the hypolipidemic properties of Echinochrome (Ech) pigment in a high-fat diet (HFD) induced hyperlipidemia in rats. After the hyperlipidemic model was set up, rats were haphazardly separated into five groups as follows: normal control group, HFD group, Atorvastatin (ATOR) group (80 mg/kg), Ech group (1 mg/kg) and combined group ATOR + Ech. The outcomes demonstrated that Ech improves lipid profile, liver functions, kidney functions and antioxidant markers of obese rats. The findings of the present investigation indicated that the Ech possesses hypolipidemic potential in obese rats.

## Introduction

Cardiovascular infirmities consolidate a wide scope of the risky issue, for instance, atherosclerosis, arteriosclerosis, stroke, and obesity [1]. Obesity has been related to an expanded danger of a progression of illnesses that include various organ-frameworks of the body [2]. Dyslipidemia is the most real and huge peril factor of a high-fat diet (HFD) intake on health [3]. Hyperlipidemia is prevalent among patients with Non-alcoholic fatty liver disease (NAFLD) [4]. The pathogenesis of NAFLD relies upon the collection of triglyceride inside hepatocytes and lipotoxicity in view of oxidative stress inside hepatocytes [5]. Oxidative stress is one of the perpetual issues in patients with hyperlipidemia [6]. Hyperlipidemia is associated with the formation of oxidized low-density lipoprotein (ox-LDL) which increases the risk of coronary artery disease, type 2 diabetes mellitus, and atherosclerosis. LDL-C is the most important target in the therapy of dyslipidemia.

Even though the fact that hypolipidemic medications can treat hyperlipidemia, they are constrained because of the absence of adequacy and safety. Statins or 3-hydroxy-3-methylglutaryl-coenzyme A reductase inhibitors (HMG-CoA reductase inhibitors) are utilized clinically for hypercholesterolemic patients with moderate and high cardiovascular illness hazard [7]. They debilitate hepatocellular cholesterol creation by inhibiting the blend of mevalonate, a critical middle person item in the cholesterol pathway [8]. Although, statins remain the standard treatment, an effective result is accomplished in just 40% of patients [9]. The greater parts of patients treated with statins are diligently exposed to skeletal muscle abnormalities, which can run from kind myalgia to serious myopathy [10].

The marine environment is a bounteous wellspring of unfamiliar utilitarian nourishments promoting the improvement of new bioactive particles with various properties [11]. For instance, sea urchins are a source of pharmacologically significant quinone pigments specifically echinochrome (Ech) and the spinochromes that constitutes a group of polyketide compounds. Emerging evidence suggests that Ech A, like many marine secondary metabolites, possess highly effective antioxidant mechanisms, including the scavenging of active oxygen radicals [12], interaction with lipoperoxide radicals [13] and inhibition of lipid peroxidation [14].

The present examination evaluated the hypolipidemic effect of echinochrome in correlation with statins in a model of high-fat diet incited hyperlipidemia in the male albino rats.

## Results

**Fig. 1:**
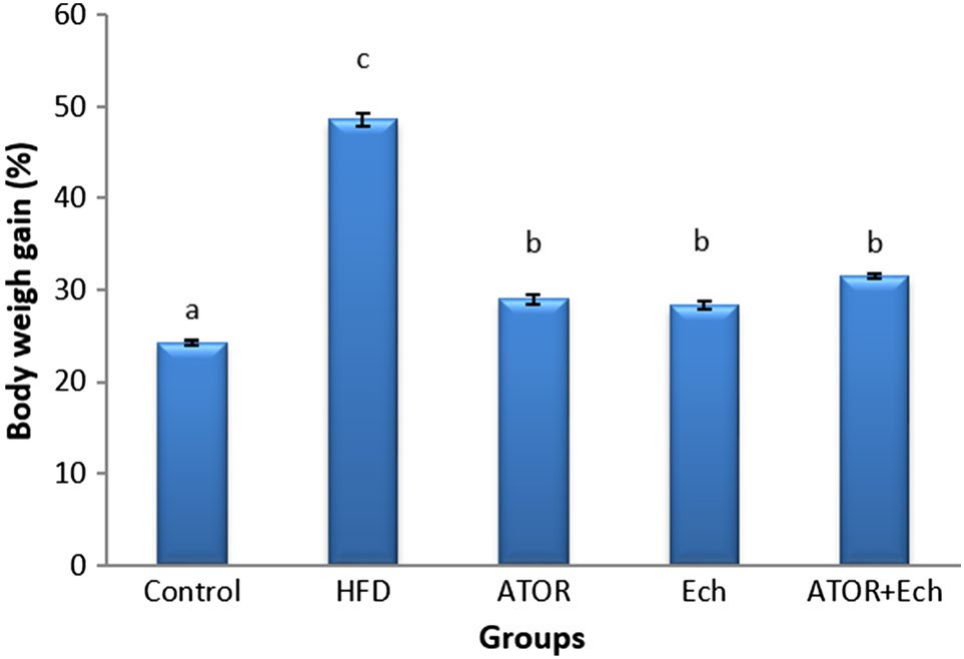
Effect of Ech and ATOR on body weight gain in hyperlipidemic rats

Figure 1 illustrates the significant elevation (p < 0.05) in weight gain after feeding with HFD for 28 days. Administration ATOR and/or Ech to obese rats reduced the body weight gain significantly.

Obesity-induced by HFD had a substantial elevation in adiposity parameters, TL, TC, TG, and LDL-C while HDL-C decreased when compared to control rats (Table 1). In contrast, the ATOR and/or Ech administration ameliorate these changes in lipid profile parameters.

**Table 1:** Effect of Ech and ATOR on lipid profile in hyperlipidemic rats

Groups	TG (mg/dl)	TL (mg/dl)	TC (mg/dl)	LDL-C (mg/dl)	HDL-C (mg/dl)
Control	17.62 ± 1.01^a^	59.50 ± 6.15^a^	114.53 ± 4.37^a^	79.87 ± 2.83^a^	39.33 ± 3.87^b^
HFD	44.73 ± 2.78^c^	272.0 ± 10.33^e^	184.67 ± 6.13^c^	166.62 ± 3.11^c^	18.83 ± 0.48^a^
ATOR	28.87 ± 0.98^b^	194.17 ± 6.24^d^	116.80 ± 2.64^a^	77.02 ± 02.80^a^	35.50 ± 1.52^b^
Ech	20.67 ± 0.83^a^	92.00 ± 11.24^b^	121.45 ± 2.77^a^	98.62 ± 1.47^b^	37.67 ± 2.16^b^
ATOR+Ech	28.78 ± 1.70^b^	143.5 ± 21.61^c^	134.17 ± 2.73^b^	93.23 ± 2.69^b^	36.17 ± 1.28^b^

Values are means ± se (n = 6 per group). Each value not sharing a common letter superscript is significantly different (P < 0.05)

Obese rats presented significant elevation (p < 0.05) in liver enzymes activities; AST, ALT, and ALP, while total proteins and albumin synthesis inhabited as compared to the control group. These changes in liver function parameters were improved after the administration of ATOR and/or Ech (Table 2).

**Table 2:** Effect of Ech and ATOR on liver biomarkers in hyperlipidemic rats

Groups	ALT (IU/ml)	ALP (IU/L)	AST (IU/ml)	T.Protein (g/dl)	Albumin (g/dl)
Control	33.17 ± 1.05^a^	67.33 ± 4.19^a^	103.83 ± 1.40^a^	6.45 ± 0.39^a^	3.75 ± 0.16^a^
HFD	54.17 ± 1.28^c^	155.33 ± 8.32^c^	197.50 ± 4.51^d^	5.70 ± 0.04^a^	3.23 ± 0.03^a^
ATOR	36.83 ± 1.96^b^	71.33 ± 4.56^b^	133.33 ± 2.85^c^	5.80 ± 0.09^a^	3.30 ± 0.07^a^
Ech	37.50 ± 2.63^b^	69.00 ± 5.57^a^	113.67 ± 4.17^b^	5.52 ± 0.09^a^	3.28 ± 0.06^a^
ATOR+Ech	37.33 ± 1.41^b^	82.67 ± 9.17 ^b^	138.83 ± 9.81^c^	5.57 ± 0.11^a^	3.32 ± 0.08^a^

Values are means ± se (n = 6 per group). Each value not sharing a common letter superscript is significantly different (P < 0.05)

HFD induced kidney dysfunction, which confirmed by the significant increase (p < 0.05) in urea, creatinine, uric acid, and CK concentrations (Table 3. While the treatment with Ech or ATOR/Ech reduced this elevation in kidney function parameters.

**Table 3:** Effect of Ech and ATOR on kidney and muscle biomarkers in hyperlipidemic rats

Groups	Creatinine (mg/dl)	Urea (mg/dl)	Uric acid (mg/dl)	Creatinine kinase (IU/l)
Control	0.40 ± 0.01^a^	28.43 ± 2.81^a^	1.18 ± 0.02^a^	103.50 ± 16.01^a^
HFD	0.76 ± 0.02^c^	64.03 ± 5.47^d^	1.58 ± 0.10^c^	236.00 ± 6.85^c^
ATOR	0.51 ± 0.02^b^	41.30 ± 7.25^c^	1.47 ± 0.02^b^	220.17 ± 15.37^c^
Ech	0.46 ± 0.01^b^	35.73 ± 1.70^b^	1.41 ± 0.04^b^	135.33 ± 21.92^b^
ATOR+Ech	0.55 ± 0.02^b^	39.85 ± 3.80^c^	1.44 ± 0.07^b^	119.33 ± 5.79^b^

Values are means ± se (n = 6 per group). Each value not sharing a common letter superscript is significantly different (P <0.05)

Compare with the control group, HFD caused a significant increase (p < 0.05) in tissues MDA concentration while GSH, GST, and CAT levels decreased significantly. Theses parameters showed no differences between the ATOR and HFD groups. While Ech alone or combined with ATOR induced a significant increase in antioxidant markers (GSH, GST, CAT) and reduced lipid peroxidation marker (MDA) (Tables 4, 5, 6).

**Table 4:** Effect of Ech and ATOR on liver oxidative stress biomarkers in hyperlipidemic rats

Groups	MDA (nmol/g tissue)	GSH (mg/g tissue)	GST (µmol/g tissue min)	CAT (U/min)
Control	2.00 ± 0.316 ^a^	6.16 ± 0.02^c^	0.5763 ± 0.03^c^	35.40 ± 3.043^c^
HFD	5.22 ± 0.267^c^	2.97 ± 0.01^a^	0.3053 ± 0.04^a^	12.60 ± 2.064^a^
ATOR	4.98 ± 0.419^c^	3.19 ± 0.04^a^	0.3378 ± 0.01^a^	15.40 ± 1.435^a^
Ech	3.66 ± 0.175^b^	5.0 ± 0.01^b^	0.4308 ± 0.03^b^	29.40 ± 2.482^b^
ATOR+Ech	3.76 ± 0.279^b^	4.79 ± 0.02^b^	0.4875 ± 0.01^b^	26.00 ± 1.048^b^

Values are means ± se (n = 6 per group). Each value not sharing a common letter superscript is significantly different (P <0.05)

**Table 5:** Effect of Ech and ATOR on kidney oxidative stress biomarkers in hyperlipidemic rats

Groups	MDA (nmol/g tissue)	GSH (mg/g tissue)	GST (µmol/g tissue min)	CAT (U/min)
Control	6.73 ± 0.09^a^	10.08 ± 0.03^d^	0.78 ± 0.02^c^	51.02 ± 2.75^c^
HFD	10.52 ± 0.27^d^	8.60 ± 0.02^a^	0.59 ± 0.03^a^	32.37 ± 3.70^a^
ATOR	10.23 ± 0.37^d^	8.96 ± 0.02^a^	0.61 ± 0.01^a^	36.55 ± 1.21^a^
Ech	8.53 ± 0.32^b^	9.90 ± 0.02^c^	0.70 ± 0.03^b^	45.62 ± 4.36^b^
ATOR+Ech	9.53 ± 0.23^c^	9.40 ± 0.10^b^	0.69 ± 0.03^b^	47.48 ± 0.34^b^

Values are means ± se (n = 6 per group). Each value not sharing a common letter superscript is significantly different (P <0.05)

**Table 6:** Effect of Ech and ATOR on muscle oxidative stress biomarkers in hyperlipidemic rats

Groups	MDA (nmol/g tissue)	GSH (mg/g tissue)	GST (µmol/g tissue min)	CAT (U/min)
Control	1.97 ± 0.02^a^	10.57 ± 0.02^c^	0.19 ± 0.01^c^	30.02 ± 2.92^c^
HFD	3.83 ± 0.14^d^	9.58 ± 0.01^a^	0.11 ± 0.01^a^	12.05 ± 1.90^a^
ATOR	3.57 ± 0.21^d^	9.86 ± 0.02^a^	0.10 ± 0.01^a^	14.62 ± 1.50^a^
Ech	2.12 ± 0.04^b^	10.07 ± 0.02^b^	0.16 ± 0.01^b^	24.90 ± 1.06^b^
ATOR+Ech	2.17 ± 0.08^c^	10.27 ± 0.03^b^	0.16 ± 0^b^	20.70 ± 4.15^b^

Values are means ± se (n = 6 per group). Each value not sharing a common letter superscript is significantly different (P < 0.05)

**Fig. 2:**
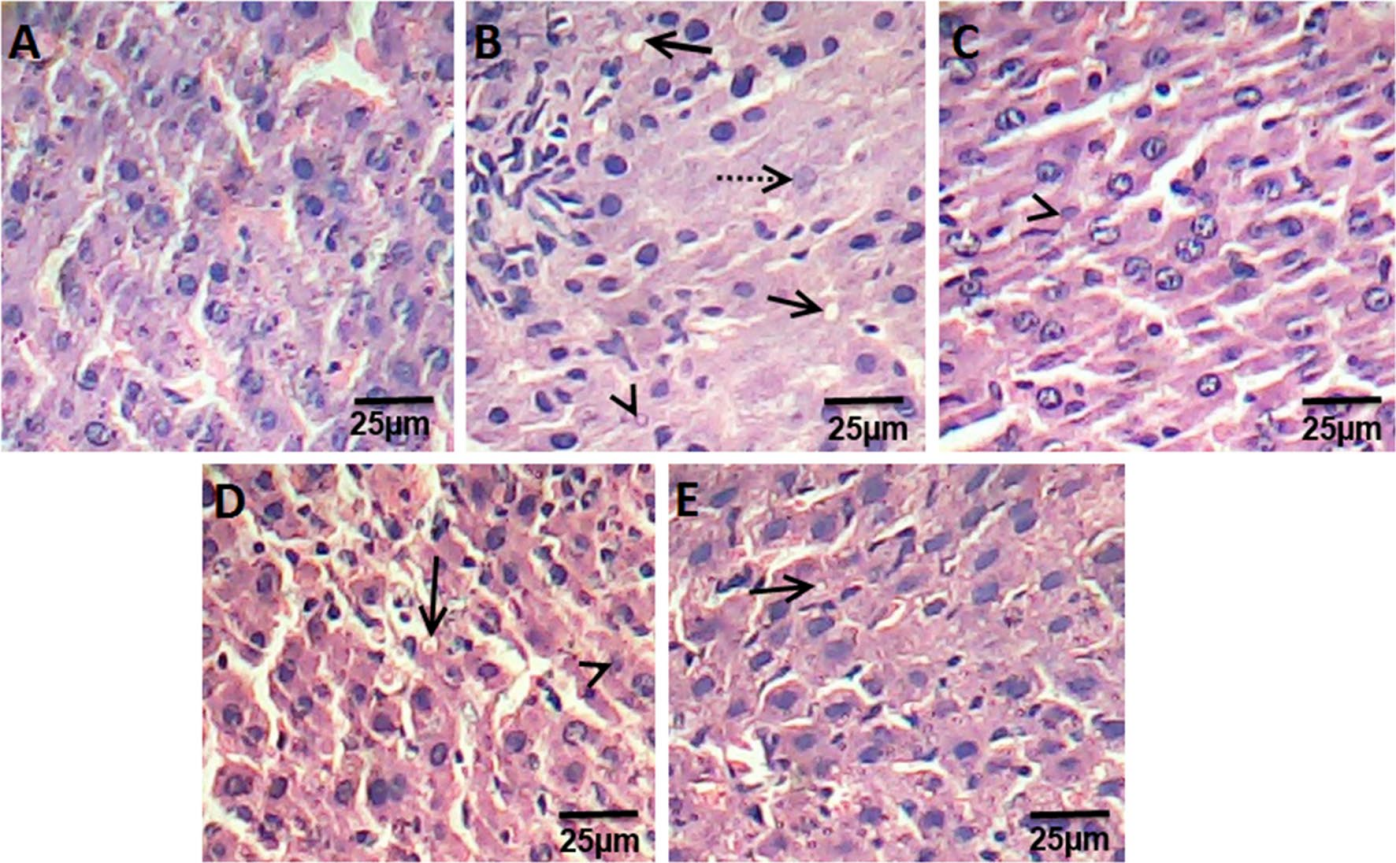
Photomicrograph of hematoxylin and eosin stained liver sections from control rats group (**a**), HFD group (**b**), ATOR group (**c**), Ech group (**d**) and ATOR + Ech group (**e**) (H&E × 400). Solid arrow: fat droplets, dotted arrow: fibrosis, arrowhead: necrosis

The liver sections of control rats are formed of the classic hepatic lobules. (Fig. 2a). The histology of liver sections obtained from HFD rats revealed clear diffused hepatic steatosis with the loss of usual concentric arrangements of hepatocytes, fatty changes, and necrosis (Fig. 2b). Liver sections of ATOR, Ech and ATOR + Ech rats showed moderate to mild degenerated changes in hepatocytes and a clear improvement in the hepatic architecture compared to HFD group (Fig. 2c–e).

**Fig. 3:**
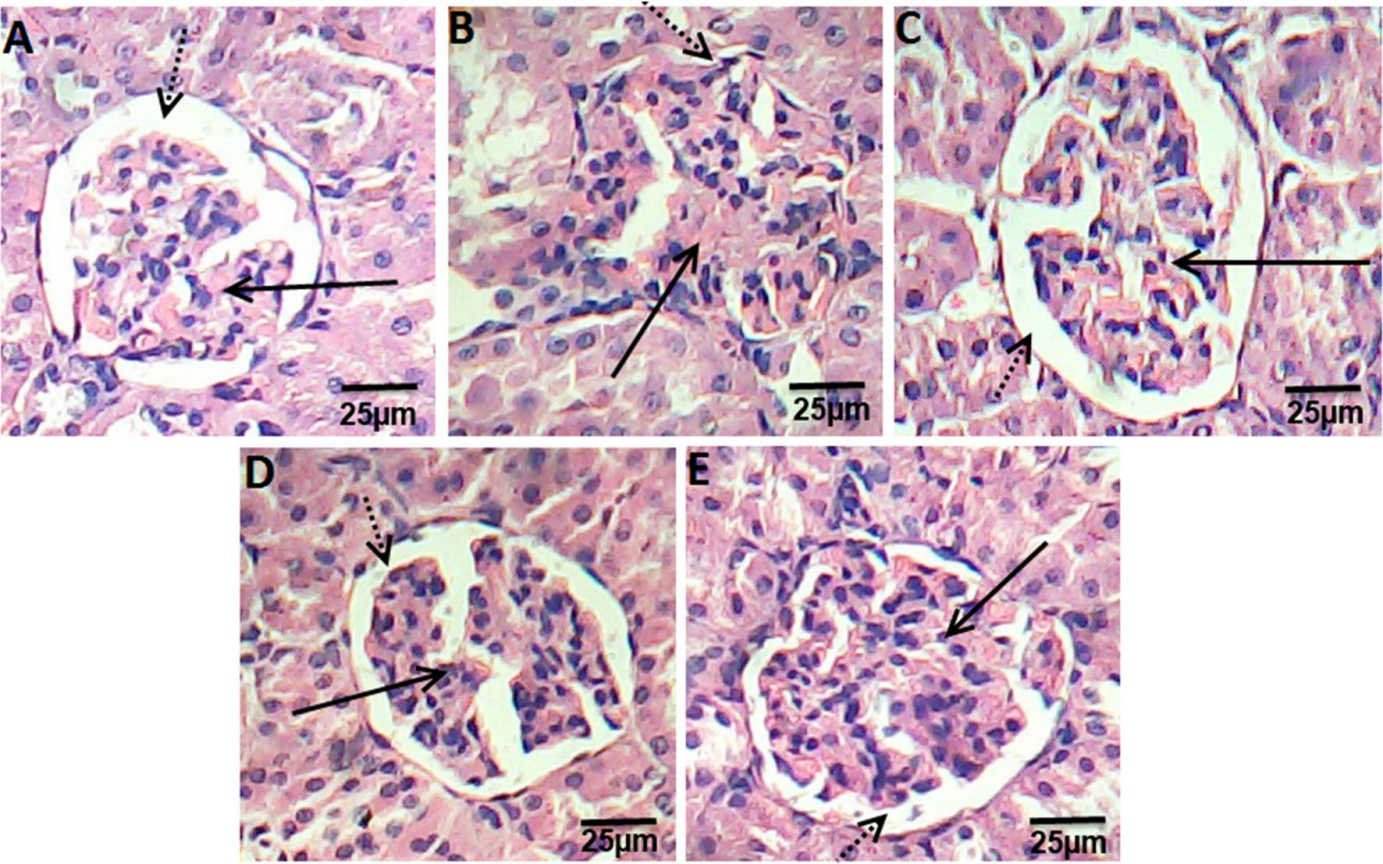
Photomicrograph of hematoxylin and eosin stained kidney sections from control rats group (**a**), HFD group (**b**), ATOR group (**c**), Ech group (**d**) and ATOR + Ech group (**e**) (H&E × 400). Solid arrow: glomeruli, dotted arrow: urinary space

Kidney sections of control groups displayed the normal appearance of the tissue where glomeruli appear as dense tufts of capillaries enclosed in the outer layer of Bowman capsules. Many renal tubules were observed (Fig. 3a). Kidney section of HFD rats presented swelling and proliferation in the endothelial cells lining the glomerular tuft of the glomeruli as well as degeneration in the epithelial cells lining the tubules, and deformed renal tissue architecture (Fig. 3a). On other hand, rats treated with ATOR, Ech, and ATOR + Ech showed mild degeneration in renal tissue architecture, as compared to HFD groups (Fig. 3c–e).

**Fig. 4:**
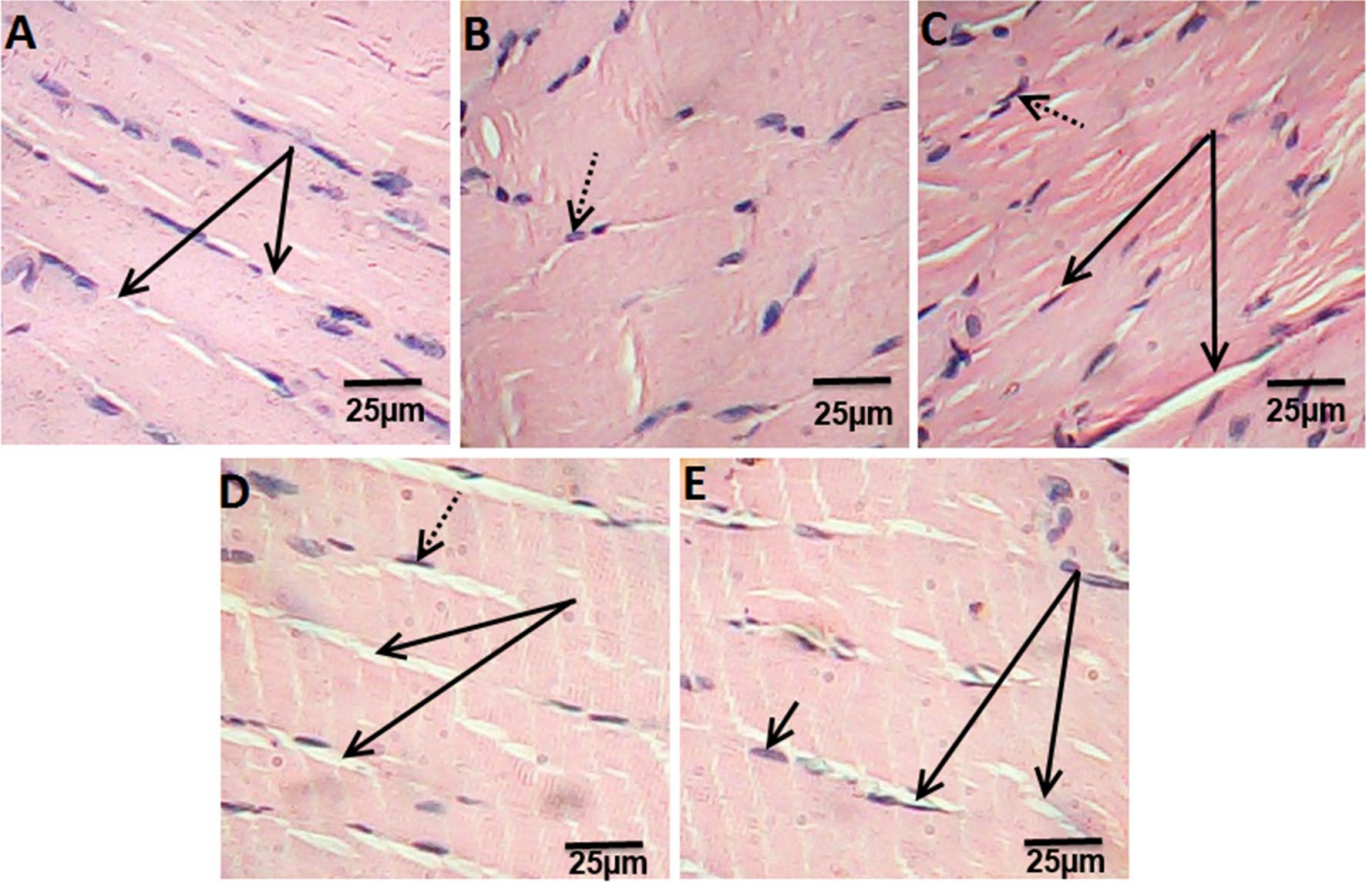
Photomicrograph of hematoxylin and eosin stained muscle sections from control rats group (**a**), HFD group (**b**), ATOR group (**c**), Ech group (**d**) and ATOR + Ech group (**e**) (H&E × 400). Solid arrow: muscle fibers striation, dotted arrow: nucleus

Histological examination of extensor digitorum longus muscle of control rats showed the normal structure of skeletal muscle (Fig. 4a). Muscle sections in HFD rats showing the splitting of the myofibers, peripheral elongated nuclei disappeared and striation is lost in some muscle fibers (Fig. 4b). While, muscle section of ATOR rats showed mild focal changes, irregular change in muscle fibers size. Nuclei were internal in position instead of peripheral and some appeared rounded in shape instead of oval. Splitting of some fibers was also observed which appeared as a transverse invagination or complete separation (Fig. 4c). Ech and ATOR + Ech treated-rats sections were associated with normalization of skeletal muscle features which were similar to that of untreated controls (Fig. 4d, e). (H & E × 400).

## Discussion

It is prominent that dyslipidemia is the most real and huge danger factor of a high-fat diet (HFD) intake on health [3]. In the present assessment, dyslipidemia was expressed in the blood of rats by increased total lipids (TL), total cholesterol (TC), low-density lipoprotein cholesterol (LDL-C), and triglycerides (TG). The histological assessments of the liver of HFD-fed rats revealed clear diffused hepatic steatosis with the loss of usual concentric arrangements of hepatocytes, fatty changes, and necrosis. Ech as well as ATOR altogether standardized the lipid profile and lessen body loads gain. It can be noticed that the hypolipidemic efficacy of Ech alone was more pronounced as compared to its combination with ATOR or ATOR alone. The hypolipidemic effect of Ech may be through its inhibition of cholesterol and triglyceride synthesis key enzymes [15].

In the present examination, HFD caused increments in concentrations of AST, ALT, and ALP enzymes and decrease total proteins and albumin concentrations. An increase in liver enzymes activity may be indicative of some liver impairment, or possibly damage [16]. Besides, the accumulation of triglyceride inside hepatocytes provoked lipotoxicity because of oxidative stress inside hepatocytes [2]. Treatment with Ech and/or ATOR caused a reduction in the activity of the liver enzymes, which may be due to its capacity to balance lipid peroxidation and heal the damaged cells [17].

Dyslipidemia appears to play a pathogenic role in the development of renal diseases [18]. In the present examination, HFD incited renal damage, which demonstrated by the height of urea, creatinine and uric acid concentrations and affirmed by the histopathological examination. Additionally, Hattori et al. [19] presumed that HFD causes macrophage infiltration in kidney coming about in glomerulosclerosis. In consonance with the finding of Yang et al. [20], the present study showed a significant decrease in the serum creatinine, urea and uric acid following treatment with Ech and/or ATOR that may be a contributory self-healing mechanism restoring the kidney structure and function.

Muscle harmed in the present assessment was verbalized in HFD rats by ahuge increment in CK and histopathological examination. It was accounted for the uncontrolled utilization of high fat-diet associated with skeletal muscle dysfunction and the development of muscle atrophy [21]. Moreover, obesity is associated with skeletal muscle loss and dysfunction and the development of muscle atrophy [21]. Also, ATOR instigated muscle damage in the present investigation. Development of myopathy could be attributed to decreased Coenzyme Q10 in muscular tissue [22]. In addition, ATOR induced oxidative stress in different tissues by inhibition of Coenzyme Q10 [23], since coenzyme Q10 has powerful antioxidant activity [24]. Then again, treatment with Ech standardized the skeletal muscle highlights close to the ordinary architecture. These results were affirmed by the histopathological assessment of the muscle, which uncovered that the treatment with Echameliorates the undesirable effect of hyperlipidemia.

Our study indicated the development of oxidative stress in the liver, kidney, and muscle of HFD- fed group. HFD can cause increased lipid peroxidation via progressive and cumulative cell injury resulting from the pressure of the large body weight [25]. In the present study, marked inhibition of lipid peroxidation and enhancement of antioxidant system (GSH, GST, and CAT) in liver, kidney and muscle tissue were evident. Nevertheless, enhanced antioxidant capacity in conjunction with reduced lipid peroxidation was observed in Ech-treated group. These results indicate the ROS scavenging activity of Ech [12]. Ech pigments have high antioxidant activity and can act by several antioxidant mechanisms, including the scavenging of active oxygen radicals [14], chelation of metal ions [26] and inhibition of lipid peroxidation [27].

Overall, our investigation exhibited the possibilities of echinochrome pigment in ameliorating hyperlipidemia induced by HFD and standardized the biochemical and histopathological changes in the liver, kidney, and muscle. Ech pigment has hypolipidemic property and antioxidant role, which reduced HFD complications in the liver, kidney, and muscles.

## General Experimental Procedures

### Chemicals and Reagents

Atorvastatin powder, purity 98.7%, purchased from SIGMA Pharmaceutical Company, Egypt. Carboxymethyl cellulose (CMC) purchased as a powder from El-Nasr Pharmaceutical Chemicals Company, Egypt.

### Echinochrome (Ech) Extraction

Sea urchins (Paracentrotus lividus) were collected from the Mediterranean shoreline of Alexandria (Egypt) at that point shipped to the research facility stuffed in ice. The collected Sea urchins were quickly shade-dried. After the evacuation of the inner tissues, the shells and spines were washed with cold water, air-dried at 4 °C for 24 h in the dark and then were grounded. The powders (10 g) were dissolved by gradually adding 20 ml of 6 M HCl. The pigments were extracted 3 times with a similar volume of diethyl ether. From that point onward, the gathered layer of ether was washed by utilizing NaCl (5%) until the acid was nearly removed. At that point, we utilized the anhydrous sodium sulfate over the solution of ether-including pigments for drying which followed by the evaporation of the solvent under reduced pressure. The extract including the polyhydroxylated naphthoquinone pigment was stored at -30°C in the dark [28, 29].

### Experimental Animals

Adult male albino rats weighing 150 ± 10 g were utilized in this investigation. The rodents were acquired from the National Organization for Drug Control and Research (NODCAR, Giza). They were housed in polyacrylic cages in the well-ventilated animal house of the Zoology Department, Faculty of Science, Cairo University. Rats were maintained in a friendly environment of a 12 h/12 h light–dark cycle at room temperature (22–25 °C) where food and water ad libitum were provided. They were acclimatized to research center conditions for 7 days before initiation of the analysis.

### Induction of Hyperlipidemia

The animals were fed a high-fat diet (HFD) with the vitality of 6.3 kcal/g, involving 19% from protein, 35% calories from fat, and 46% from sugar for four weeks [30]. The eating regimen comprises of a blend of 69% ordinary chow pellet (bought from El Gomhorya Company, Ismailia, Egypt), 6% corn oil, 19% milk powder, and 6% ghee. While, the typical rodent chow diet contains 3.16 kcal/g with 21% from protein, 48.8% from sugar and 3% from fat.

### Experimental Plan

Thirty rats were assigned into five main groups (6 rats/group).

#### Control Group

Rats were fed normal diets for 4 weeks, then given orally 0.5% CMC for 16 consecutive days.

#### HFD Group

Rats were fed HFD for 4 weeks, and then were given 0.5% CMC orally for 16 days.

#### Atorvastatin (ATOR) Group

Rats were fed HFD for 4 weeks, then given orally ATOR(80 mg/kg in 0.5% CMC) [31] daily for 16 days.

#### Ech Group

Rats were fed HFD for 4 weeks, then given orallyEch(1mg/kg in 0.5% CMC) [32] daily for 16 days.

#### Ator + Ech Group

Rats were fed HFD for 4 weeks, then given orallyATOR and after one hour were given orally Ech at the same doses and duration as ATOR and Ech groups.

### Animal Handling

At the end of the experiment, rats were euthanized with an overdose of sodium pentobarbital (100 mg/kg). The blood collected from the rats via cardiac puncture and then was separated by centrifugation (3000 rpm, 15 min) to obtain sera which were stored at − 80 °C for the biochemical measurements. The liver, kidney, and muscle were removed and were immediately blotted using a filter paper to remove traces of blood. Portions of these tissues were put away at − 80 °C for biochemical examination. Different pieces of liver and kidney tissues were suspended in 10% formal saline for fixation preparatory to histopathological assessment.

### Tissue Homogenate Preparation

The liver, kidney, and muscle tissues were homogenized (10% w/v) in ice-cold 0.1 M Tris–HCl buffers (pH 7.4). The homogenate was centrifuged at 860×*g* for 15 min at 4 °C and the resultant supernatants were used for the oxidative stress analyses.

### Biochemical Assays

The serum aspartate aminotransferase (AST) and alanine aminotransferase (ALT) were estimated by the method of Reitman and Frankel [33], serum alkaline phosphatase (ALP) [34], serum total protein [35], serum albumin [36], serum total lipids (TL) [37], serum triglycerides (TG) [38], serum total cholesterol (TC) [39], serum low density lipoprotein (LDL-C) [40] and serum high density lipoprotein (HDL-C) [41], serum creatinine [42], serum urea [43], serum uric acid [44], creatine kinase (CK) [45], MDA [46], glutathione reduced (GSH) [47], glutathione-S-transferase (GST) [48], and catalase [49] according to the manufactures instructions using Biodiagnostic kits (Giza, Egypt).

### Histopathological Examination

The liver, kidney and muscle tissues were removed immediately, washed and fixed in neutral buffered formalin (10%) for further processing by the ordinary routine work: dehydration, clearing, and embedding. The paraffin-embedded blocks of the liver and kidney tissues were cut by using microtome in 4 μm-thick tissue sections then hematoxylin and eosin were used for staining. The tissue sections were assessed under light microscopy independently by 2 investigators in a blinded way.

### Statistical Analysis

Data of the current study are represented in tables as mean ± SE and were analyzed by one-way ANOVA followed by Duncan post-hoc test for multiple comparisons using the Statistical Package for the Social Sciences software (SPSS 20). The differences between means were considered statistically significant when the P value was less than 0.05.
